# *Listeria ivanovii*—an underestimated pathogen in veterinary medicine

**DOI:** 10.3389/fvets.2026.1844936

**Published:** 2026-05-28

**Authors:** Tamara Kozytska, Heinrich Neubauer, Gamal Wareth

**Affiliations:** 1 Institute of Bacterial Infections and Zoonoses, Friedrich-Loeffler-Institut, Jena, Germany; 2 Department of Bacteriology, Immunology, and Mycology, Faculty of Veterinary Medicine, Benha University, Toukh, Egypt

**Keywords:** abortion, animal listeriosis, *Listeria ivanovii*, ruminants, veterinary diagnostics

## Abstract

*Listeria* (*L.*) *ivanovii* is a Gram-positive, facultatively intracellular rod-shaped bacterium. It is predominantly associated with animal infections, particularly in ruminants. Unlike *L. monocytogenes*, which is widely recognised as a major zoonotic and foodborne pathogen, *L. ivanovii* is often overlooked, despite documented cases of abortion, stillbirth, and neonatal septicemia in sheep, goats, and cattle. This review provides an overview of *L. ivanovii* taxonomy, microbiological characteristics, virulence determinants, host adaptation, epidemiology, transmission routes, diagnostic challenges, antimicrobial susceptibility, and preventive measures in animal husbandr*y*. Available evidence suggests that infections with *L. ivanovii* tend to be sporadic, with occasional outbreaks of abortions in small ruminant flocks. Large-scale epizootics and sustained interregional spread have not been documented. Human infections are rare and predominantly occur in immunocompromised individuals, which supports the assumption that the zoonotic potential is limited under normal conditions. Underdiagnosing and missing awareness likely contribute to the perception of rarity, as routine laboratory workflows often do not differentiate *Listeria* species beyond the genus level. The increasing use of molecular confirmation and whole-genome sequencing may improve species-level detection and clarify epidemiological patterns. Overall, *L. ivanovii* should be regarded as a specialised pathogen predominantly associated with ruminants and of veterinary relevance. Improved diagnostics and integration into existing surveillance frameworks could enhance our understanding of its true epidemiological role and prevent it from being endemic.

## 1 Introduction

The genus *Listeria* (*L.*) comprises a diverse group of Gram-positive bacteria found in a variety of environments, including soil, water, vegetation, and food processing settings. While most species are non-pathogenic, a few are of significant clinical and veterinary importance. Of the recognised species, *L. monocytogenes* has attracted most attention from scientists and clinicians due to its important role as a zoonotic pathogen, which is mainly transmitted through food, the placenta (from mother to foetus), and direct contact with animals (1, 2). In contrast, *L. ivanovii* remains relatively understudied despite its well-documented association with severe disease in animals, particularly in ruminants (3–6). *L. ivanovii* is currently divided into two recognised subspecies, *L. ivanovii* subsp. *ivanovii* and *L. ivanovii* subsp. *londoniensis*, based on genetic and phenotypic differences. The nominative subspecies, *L. ivanovii* subsp. *ivanovii* is more commonly associated with animal infections, whereas *L. ivanovii* subsp. *londoniensis* is less frequently reported and remains comparatively understudied (7, 8).

*L. ivanovii* was first described as species of its own due to its biochemical properties and host specificity. Early studies indicated a clear preference for animals as primary hosts rather than humans. This highlights the genetic and phenotypic independence of the species, as *L. monocytogenes* has a broad host range. *L. ivanovii* is primarily adapted to domestic livestock, particularly sheep, goats, and cattle (3, 4, 9).

The literature shows that *L. ivanovii* primarily affects ruminants, particularly sheep and goats. In these animals, it is mainly associated with reproductive disorders and systemic infections, especially in young animals. Cases of visceral infection and septicaemia have been reported in weaned lambs, suggesting that, under certain circumstances, the bacterium can cause severe invasive diseases (10). Although rare, infections have also been reported in atypical hosts, e.g., cats (11).

Routine diagnostic procedures in some laboratories may identify *Listeria* isolates only to the genus level, and species-level differentiation is not always performed (12). In addition, phenotypic similarity and overlapping biochemical profiles can occasionally lead to misidentification of *L. ivanovii* as *L. monocytogenes*, particularly in the absence of molecular confirmation or optimised MALDI-TOF MS databases (12, 13). Therefore, it is possible that the true number of *L. ivanovii* infections is underestimated in certain contexts; likely available data remain limited and are largely based on sporadic case reports.

At the molecular level, *L. ivanovii* possesses a unique set of virulence factors, including ivanovin O and sphingomyelinase C (SmcL), that differ from those of *L. monocytogenes*. These factors are believed to contribute to host specificity and tissue tropism (14, 15). Genomic analyses further support the idea that *L. ivanovii* is an animal-adapted pathogen, with virulence mechanisms optimised for infecting ruminant hosts rather than for efficient transmission to humans (8, 16).

Infections in humans caused by *L. ivanovii* are rarely reported and are typically linked to severe immunosuppression, suggesting that it has limited zoonotic potential under normal circumstances (17). Although *L. ivanovii* is occasionally found in food products or environmental samples, it is primarily relevant to animal health rather than public health (1, 17).

By summarising current knowledge and identifying critical gaps, this review aims to raise awareness of *L. ivanovii* as a veterinary pathogen and encourage research and the amendment of diagnostic practise. This review summarises current knowledge of *L. ivanovii* infection in animals, emphasising clinical relevance and identifying existing knowledge gaps.

## 2 Taxonomy and microbiological characteristics

*L. ivanovii* belongs to the genus *Listeria* within the family *Listeriaceae* and the phylum *Firmicutes* (*Bacillota*). According to the current taxonomy as provided by the List of Prokaryotic Names with Standing in Nomenclature (LPSN) database, the genus *Listeria* includes 29 species-level entries at the time of writing^1^ (18, 19). Genus *Listeria* comprises Gram-positive, facultative intracellular bacteria that are widespread in the environment and can infect animals and humans (6, 7, 20–22). *L. ivanovii* was formally described as a species of its own in 1984 and was initially considered primarily an animal-associated pathogen, particularly affecting ruminants (22, 23). Subsequent taxonomic and molecular studies have identified two subspecies: *L. ivanovii* subsp. *ivanovii* and *L. ivanovii* subsp. *londoniensis* (7, 24). The nominative subspecies, *L. ivanovii* subsp. *ivanovii* is well-characterised and frequently reported from veterinary cases (25). Morphologically, *L. ivanovii* is a Gram-positive, non-spore-forming, motile rod, typically measuring approximately 0.4–0.5 μm in width and 1–2 μm in length. Motility is mediated by peritrichous flagella and is usually observed at lower temperatures, particularly around 20–25 °C (20). Like other members of the genus, the bacterium is catalase-positive, oxidase-negative, and capable of facultative anaerobic growth (26, 27). The species exhibits considerable environmental adaptability, growing over a wide temperature range of approximately 4–45 °C. This enables it to survive in a variety of environments, including soil, water, animal housing, and feed materials (22, 26, 28).

Colonies of *L. ivanovii* typically appear after 24–48 h of incubation at 35–37 °C on non-selective nutrient media such as nutrient agar or tryptic soy agar (TSA) as small (1–2 mm), smooth, greyish-white colonies with well-defined margins (22, 29). When cultured on blood agar, typically supplemented with 5% defibrinated sheep blood, *L. ivanovii* produces characteristic *β*-hemolysis, often more pronounced than that observed with *L. monocytogenes* (22). For the selective isolation of *L. ivanovii* from clinical or environmental material, particularly during investigations into abortions in ruminants, standard *Listeria* selective media such as Oxford, PALCAM agar, or chromogenic media such as ALOA agar (Ottaviani & Agosti) are commonly used (22, 30). However, these media only permit genus-level detection and cannot differentiate between *Listeria* spp. (6). Only ALOA agar allows identification of the pathogenic species *L. monocytogenes* and *L. ivanovii*, but not between them (30).

In cases where bacterial concentrations are low or samples contain competing microflora, a selective enrichment step using enrichment broths, such as Half-Fraser (primary enrichment – allows for the recovery of stressed *Listeria* cells) and Fraser broths (secondary enrichment – allows for the suppression of background flora and may increase isolation sensitivity), can improve isolation sensitivity (29, 30). As phenotypic characteristics can overlap amongst species within a genus, identification at the species level requires additional biochemical, molecular, or proteomic methods, such as PCR, sequencing, or MALDI-TOF mass spectrometry, with relevant reference databases (26). One classical phenotypic method that is useful for differentiating *L. ivanovii* from other *Listeria* species is the CAMP test. In this assay, *L. ivanovii* typically produces a positive CAMP reaction with *Rhodococcus equi*, whereas *L. monocytogenes* usually shows CAMP positivity with *Staphylococcus aureus* (20, 21).

Additional biochemical characteristics used in routine differentiation include carbohydrate fermentation patterns. For instance, *L. monocytogenes* usually ferments rhamnose but not xylose, whereas *L. ivanovii* generally exhibits the opposite behaviour (20, 26, 31, 32). Table 1 summarises the most important biochemical and phenotypic traits used to differentiate between *L. monocytogenes* and *L. ivanovii* in routine microbiological diagnostics.

**Table 1:** Biochemical and phenotypic differentiation of *L. monocytogenes* and *L. ivanovii.*

Biochemical characteristic	*L. monocytogenes*	*L. ivanovii*	Diagnostic significance	Reference
*β* Hemolysis on blood agar	Moderate *β*-hemolysis	Stronger *β*-hemolysis	Species differentiation	(22)
CAMP test (*Staphylococcus aureus*)	Positive	Negative	Species differentiation	(21)
CAMP test (*Rhodococcus equi*)	Negative	Positive	Species differentiation	(21)
Rhamnose fermentation	Positive	Negative	Species differentiation	(32)
Xylose fermentation	Negative	Positive	Species differentiation	(32)
Mannitol fermentation	Negative	Negative	Genus identification	(22)
Catalase test	Positive	Positive	Genus identification	(32)
Oxidase test	Negative	Negative	Genus identification	(32)
Esculin hydrolysis	Positive	Positive	Genus identification	(21)
Motility (umbrella test, 25 °C)	Positive	Positive	Limited diagnostic value	(22)
H2S production	Negative	Negative	Limited diagnostic value	(20)

## 3 Virulence factors and pathogenesis

The virulence profile of *L. ivanovii* differs from that of *L. monocytogenes,* reflecting its evolutionary position within the genus and its apparent adaptation to animal hosts. In addition to LIPI-2, *L. ivanovii* harbours LIPI-1, the core pathogenicity island conserved across pathogenic *Listeria* species, which encodes key virulence factors including PrfA, phospholipases (PlcA, PlcB), and proteins involved in intracellular survival and cell-to-cell spread. Recent reviews emphasise that, although *L. ivanovii* exhibits the typical intracellular lifestyle of pathogenic *Listeria*, its pathogenicity is closely linked to virulence determinants encoded in LIPI-2 (*Listeria* pathogenicity island 2). These determinants are either absent in *L. monocytogenes* or function differently (33). The main factor determining the virulence of *L. ivanovii* is ivanolisin O (ILO), a cholesterol-dependent cytolysin that is functionally similar to listeriolysin O (LLO) found in *L. monocytogenes*. ILO promotes *β*-haemolysis and plays a crucial role in membrane destruction during infection (33, 34). Studies in which LLO replaced ILO in recombinant *L. ivanovii* strains demonstrate that haemolysins are essential for intracellular survival and interaction with host cells. This further confirms the functional importance of ILO in the pathogenic cycle (34). Similar to LLO in *L. monocytogenes*, ILO is believed to enable bacteria to escape from phagosomes after they are engulfed by host cells, thereby facilitating cytosolic replication. However, structural, regulatory, and activity differences between ILO and LLO may contribute to variations in host specificity and tissue tropism (33).

One of the most distinctive features of the virulence of *L. ivanovii* is the presence of the *smc*L gene, which is located in LIPI-2 and encodes sphingomyelinase C (SmcL). SmcL hydrolyses sphingomyelin in eukaryotic membranes, thereby contributing to cytotoxicity and haemolytic activity (35).

Like other pathogenic *Listeria* species, *L. ivanovii* encodes internalin-like proteins that mediate adhesion to and invasion of host cells. Amongst these proteins, InlB2 is considered a potential contributor to host cell penetration (33). While the molecular mechanisms of InlB2 are less well characterised than those of InlA/InlB in *L. monocytogenes*, genomic data suggest that *L. ivanovii* possesses the fundamental genetic machinery required for intracellular invasion and cytosolic replication (16, 33). In addition to the determinants associated with LIPI-2, several other factors associated with virulence have been described or predicted in *L. ivanovii* (36). These include internalin homologues involved in host cell adhesion and invasion, and regulatory proteins such as *PrfA*, which plays a central role in controlling the expression of virulence genes in the genus *Listeria*. The alternative sigma factor *SigB* plays a role in stress response regulation and contributes to bacterial survival under adverse environmental and host-associated conditions (37). Furthermore, genes encoding phospholipases and stress response proteins may facilitate intracellular survival and adaptation to host environments. While the functional roles of many of these factors are less well characterised than those of *L. monocytogenes*, available genomic and experimental data suggest that *L. ivanovii* has a broader range of virulence determinants than was previously recognised (38). Table 2 presents a summary of the key virulence-associated factors of *L. ivanovii*.

**Table 2:** Virulence-associated factors of *L. ivanovii.*

Virulence factor	Gene/System	Function	Presence in *L. ivanovii*	Comparison with *L. monocytogenes*	Reference
Ivanolysin O (ILO)	*ilo*	Cholesterol-dependent cytolysin; membrane disruption; phagosomal escape	Present (LIPI-2)	Functional analogue of LLO	(21, 22)
Sphingomyelinase C	*smcL*	Sphingomyelin hydrplysis; cytotoxicity; haemolysis	Present (LIPI-2)	Absent in *L. monocytogenes*	(15, 21)
LIPI-1 pathogenicity island	LIPI-1 cluster	Core virulence island encoding *PrfA, PlcA, PlcB, ActA;* intracellular survival and cell-to-cell spread	Present	Conserved in pathogenic *Listeria*	
LIPI-2 pathogenicity island	LIPI-2 cluster	Encodes ILO, *SmcL* and associated virulence factors	Present	Absent in *L. monocytogenes*	(23, 59)
Internalin-like proteins	*inl* homologues (e.g., InlB2)	Host cell adhesion and invasion	Present	Similar but less characterised	(22)
PrfA regulator	*prfA*	Master regulator of virulence gene expression	Present	Conserved regulator	(22, 60)
Phospholipases	*plcA, plcB* homologues	Phagosomal escape; intracellular survival	Predicted / present	Conserved function	(22, 60)
Stress response proteins	*Sig σB*	Regulation of stress response; adaptation to host induced stress	Present	Conserved mechanisms	(37, 60)
Actin-based motility	*actA* homologues	Intracellular motility; cell-to-cell spread	Present	Well characterised in *L. monocytogenes*	(22, 61)

## 4 Animal hosts, epidemiology, and global distribution of *Listeria ivanovii*

In animals, infections caused by *L. ivanovii* are most commonly associated with reproductive disorders including isolated cases and outbreaks of abortion, stillbirths and neonatal septicaemia particularly in ruminants (3–5, 10). Most available evidence suggests that *L. ivanovii* primarily affects ruminants, particularly sheep and goats, with many reported cases involving reproductive disorders (3, 5, 9). Such lesions are typically characterised by multifocal necrosis, predominantly affecting the liver, spleen, and placental tissues, most often presenting as coagulative necrosis.

Several well-documented outbreaks of abortion in sheep have been associated with *L. ivanovii*, sometimes affecting multiple animals within a single flock. For example, an outbreak reported in India described the consistent isolation of *L. ivanovii* from aborted foetuses and placentas tissues, supporting its role as the causative agent in that context (5). Similar outbreaks have been described in Türkiye, where reproductive losses were observed (9, 39). *L. ivanovii* is most commonly associated with abortions, stillbirths, and the birth of weak offspring in sheep. In addition to reproductive pathology, cases of systemic infection have been reported in lambs, including visceral infection and septicemia (10). This observation suggests that *L. ivanovii* may be capable of causing invasive disease beyond the reproductive system.

In goats, available data remain limited; however, sporadic cases of abortion and systemic infection in small ruminants have been reported (40). Reports of infection in pigs are rare (41), and cases in horses have been documented only sporadically, typically in the context of broader diagnostic investigations (26).

In cattle, *L. ivanovii* has been identified in cases of abortion, although such reports remain relatively infrequent. Several studies have described isolation of the bacterium from foetal tissues, supporting its potential role in reproductive pathology in cattle (3, 4).

In addition to reproductive diseases, *L. ivanovii* has been associated with systemic infections in young animals. Dunnett et al. reported cases of visceral infection and septicaemia in weaned lambs demonstrating that under certain conditions, *L. ivanovii* can cause severe manifestations of diseases unrelated to reproductive function (10). These findings imply that the clinical spectrum of *L. ivanovii* infection in animals could encompass more than just abortion, potentially including invasive diseases in newborns and young ruminants.

There have been isolated reports of *L. ivanovii* infecting atypical hosts. Recently, the first isolation of *L. ivanovii* from tissue samples of a kitten with acute catarrhal gastroenteritis was reported. The isolated microorganism was identified as *L. ivanovii* based on its phenotypic characteristics and 16S rRNA sequencing. The subspecies *L. ivanovii* subsp*. ivanovii* was identified through comparative analysis of *sigB* gene sequences (11). A case of septicemia caused by *L. ivanovii* in a chinchilla in Belgium has also been reported (42).

While such cases remain rare, they demonstrate that the agent has the potential to cause invasive infection under certain conditions. Unlike *L. monocytogenes*, however, large-scale epizootic outbreaks in wild or exotic animals have not been described for *L. ivanovii*, which may emphasise its relative specialisation in ruminants.

**Figure 1:**
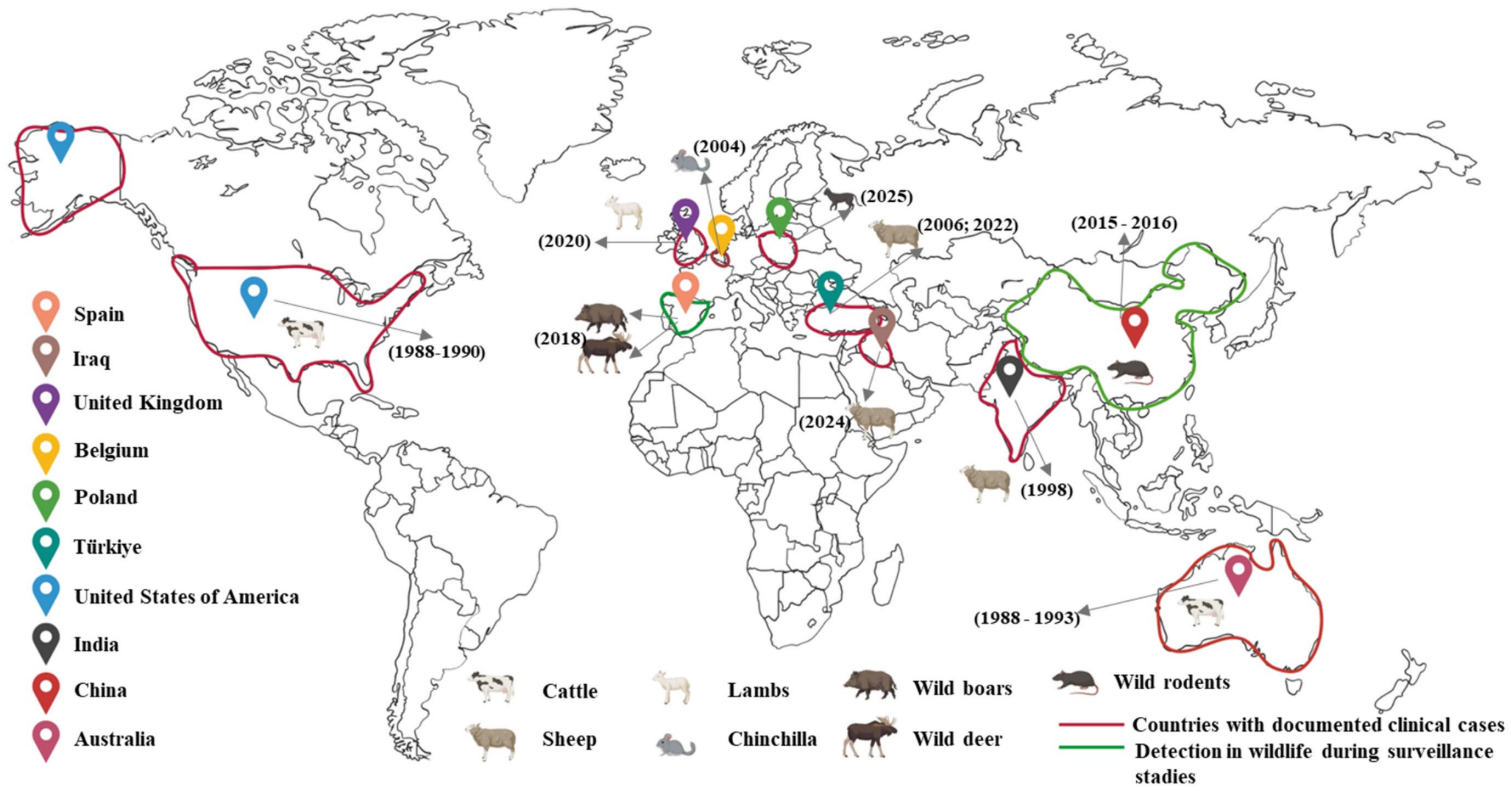
Global distribution of reported *L.ivanovii* infections and detections in animals. Countries with documented clinical cases of *L. ivanovii* infection in animals are outlined in red. Coloured markers indicate countries where clinical cases of infection in domestic animals, including sheep, cattle, lambs, chinchillas, and cats, have been reported. The green outline indicates countries where *L. ivanovii* has been detected in wild animals, including wild rodents, deer, and boar, during scientific research. The years indicate the periods during which cases or detections were reported in the literature (Table 3). Figure created in BioRender and modified in PowerPoint.

There have been reports of clinical cases of *L. ivanovii* infection from multiple geographic regions worldwide, including Europe, Asia, Australia, and North America (see Table 3; Figure 1). Most reports tend to describe isolated cases or small, localised outbreaks rather than large-scale epizootics.

**Table 3:** Documented clinical cases of *L. ivanovii* infection in animals worldwide.

Animal species	Clinical signs	Sample type	Diagnostic method	Country	Year	Reference
Sheep	Abortion	Foetuses, placenta	Culture	India	1998	(5)
Sheep	Abortion	Foetal tissues	Culture	Türkiye	2006	(9)
Sheep	Abortion	Milk, vaginal swabs, foetus	Culture, PCR, WGS	Türkiye	2022	(39)
Sheep	Abortion	Foetuses	Culture, PCR	Iraq	2024	(62)
Cattle	Abortion	Foetal tissues	Culture	USA	1988–1990	(3)
Cattle	Abortion	Foetal organs	Culture	Australia	1988–1993	(4)
Lambs (weaned)	Systemic infection	Post-mortem organs	Culture	United Kingdom	2020	(10)
Chinchilla	Systemic infection	Liver	Culture	Belgium	2004	(42)
Cat	Systemic infection	Internal organs	Culture, WGS	Poland	2025	(11)
Wild Rodents	Surveillance study	Large intestine	Culture, PCR, WGS	China	2015–2016	(63)
Wild Deer and Boars	Surveillance study	Tonsils	Culture, WGS	Spain	2018	(64)

## 5 Transmission routes and reservoirs in animal populations

The transmission of *L. ivanovii* in animal populations follows epidemiological patterns that are broadly similar to those observed for *L. monocytogenes*. However, available data remain limited. Current knowledge largely relies on case reports and investigations of localised outbreaks rather than systematic epidemiological studies (5, 26). In ruminants, the faecal–oral route is considered the primary transmission pathway. Infected animals shed *L. ivanovii* in their faeces, which can contaminate the farm environment including bedding, feed, soil, and water sources (26, 43). As with other *Listeria* species, the bacterium can persist in the environment due to its tolerance of a variety of environmental stress and its ability to grow at low temperatures (22, 26, 44).

Farm environments, therefore, represent important secondary reservoirs of the pathogen. Contaminated bedding, feeding equipment, soil, and drinking water can facilitate horizontal transmission within herds or flocks, especially in areas where lambing or calving occurs and animal density increases (5, 22). The feed itself may also represent a potential source of exposure. There is limited direct evidence linking silage contamination to *L. ivanovii* outbreaks. Hence, poorly fermented silage, spoiled feed and organic farm waste could facilitate environmental persistence and transmission (26, 45). Vertical transmission is considered an important component of the epidemiology of *L. ivanovii* infections in ruminants. Following systemic infection and bacteraemia in pregnant animals, the bacterium can spread haematogenously to the placenta resulting in placentitis and foetal infection (22). Aborted foetuses and placental tissues often harbour high bacterial loads and can heavily contaminate the environment, thereby increasing the risk of exposure for other animals in the herd or flock.

Unlike *L. monocytogenes*, which is widely distributed across diverse ecological niches, including wildlife and food-processing environments, *L. ivanovii* appears to be more strongly associated with livestock production systems (26, 44).

## 6 Diagnostics in veterinary practise

Although sensitive detection and species-level identification of *L. ivanovii* in veterinary diagnostics are technically feasible, they remain neglected and thus hinder epidemiological analyes. In investigations into ruminant abortion, conventional bacteriology still remains the cornerstone of laboratory confirmation. Isolation is typically performed using samples from aborted foetuses, i.e., placental tissue, the liver, the spleen or the abomasum. The standardised ISO 11290-1 protocols for the qualitative detection of *Listeria* spp. (i.e., presence/absence), which are widely used in food and environmental microbiology, are also suitable for recovering *Listeria* spp. in veterinary settings. These methods are based on selective enrichment using Half-Fraser and Fraser broths. These broths are specifically designed to stimulate the growth of *Listeria* spp., while simultaneously inhibiting the growth of other microflora. The methods provide preliminary detection based on the hydrolysis of esculin, followed by plating on selective agar media such as Oxford, PALCAM, or ALOA (Ottaviani & Agosti) (30, 45). On Oxford and PALCAM agar, *Listeria* colonies typically appear as small, grey-to-greenish colonies surrounded by a black halo due to esculin hydrolysis in the presence of ferric ions, whereas on ALOA medium, colonies are usually blue-green with an opaque halo resulting from phospholipase activity (21, 27). However, these media only permit genus-level detection and cannot differentiate between *Listeria* species (5).

*L. ivanovii* grows well under ISO-based conditions and forms small greyish colonies with *β*-haemolysis on blood agar. However, haemolysis does not reliably distinguish between *L. ivanovii* and *L. monocytogenes*. It is important to note that ISO 11290 is optimised for the detection of *L. monocytogenes* and *Listeria* spp., rather than for the detailed differentiation of species. Consequently, laboratories may report “*Listeria* spp. isolated” without further speciation, which likely contributes to the under-recognition of *L. ivanovii* in veterinary abortion cases (30, 35, 45).

Selective chromogenic media such as ALOA allow the presumptive differentiation of pathogenic *Listeria*, which exhibit phospholipase activity and form opaque halos around their colonies. Both *L. monocytogenes* and *L. ivanovii* typically yield positive results, so additional tests are required for species confirmation (30, 45, 46). Historically, CAMP-like synergistic haemolysis with *Rhodococcus equi* as well as sphingomyelinase C activity have been used as phenotypic markers for *L. ivanovii*. However, phenotypic variability and overlap limit the reliability of biochemical identification alone (35).

Automated biochemical systems, e.g., VITEK 2, can provide a rapid preliminary identification, but the performance for rare species depends on the completeness of the database. Misidentification of uncommon *Listeria* spp. has been reported when reference libraries are limited or spectra are suboptimal. Therefore, VITEK-based identification of *L. ivanovii* should be interpreted with caution and, when epidemiologically relevant (e.g., abortion storms), confirmed by molecular methods (12, 47). In contrast, immunoassay-based platforms such as the miniVIDAS system are primarily designed for detecting *Listeria* spp., specifically *L. monocytogenes*, in screening contexts and do not reliably provide species-level differentiation of *L. ivanovii* (30, 45). Thus, miniVIDAS should be regarded as a screening tool rather than a speciation method.

MALDI-TOF MS has become routine in many veterinary laboratories and generally enables species-level identification of *Listeria,* provided that updated reference databases are used. Earlier library versions occasionally yielded ambiguous results for rare species, but performance has improved with the expansion of the database (12). Nevertheless, confirmatory testing is recommended when unusual hosts, severe systemic disease or epidemiological investigations are involved.

Species-specific PCR assays targeting *smc*L (which encodes sphingomyelinase C in LIPI-2) can reliably distinguish *L. ivanovii* from *L. monocytogenes,* making them particularly valuable for diagnosing abortions (35, 48). Although multiplex PCR panels for abortion pathogens increasingly incorporate genus-level *Listeria* detection, primer design is crucial for achieving reliable species resolution. Comparative genomic analyses emphasise the importance of validated, species-specific targets because of the genetic diversity within *L. ivanovii* (Table 4) (33, 48).

**Table 4:** Diagnostic methods for the identification of *L. ivanovii* in veterinary practise.

Method	Target detected	Species-level identification	Strengths	Limitations	Recommended use
Classical culture (blood agar)	*Listeria* spp.	No	Simple, inexpensive	Non-specific	Initial isolation
Standardised culture methos (ISO 11290-1)	*Listeria* spp.	Limited	Standardised, widely accepted	Optimised for *L. monocytogenes*	Food, feed, and environment
CAMP test (*Rhodococcus equi*)	*L. ivanovii*	Presumptive	Simple	Variable results	Preliminary identification
Biochemical tests	*Listeria* spp.	Limited	Low cost	Variable results	Preliminary identification
VITEK 2	*Listeria* spp.	Limited	Rapid, automated	Database-dependent; rare species misidentification	Preliminary identification
Immunoassay (miniVIDAS)	*Listeria* spp. or *L. monocytogenes* antigens	No	Rapid screening	Does not reliably detect *L. ivanovii*	Screening only
MALDI-TOF MS	*Listeria* spp.	Yes	Rapid, cost-effective	Database-dependent	Routine identification (validated databases)
Species-specific PCR (*smc*L)	*L. ivanovii*	Yes	Specific and sensitive	Requires molecular setup	Confirmation
Whole-genome-sequencing	Genus, (sub)species, genotype	Yes	Highest resolution	Costs, infrastructure	Epidemiology, tracing, research

Whole-genome sequencing (WGS) is the most sensitive approach for species confirmation and subspecies differentiation (subsp. *ivanovii* vs. subsp. *londoniensis*) as well as phylogenetic analysis. Recent investigations demonstrate substantial diversity amongst isolates from animal and human sources, illustrating the epidemiological value of WGS in outbreak investigation and surveillance (33, 49). WGS is not yet universally implemented in veterinary laboratories, but decreasing sequencing costs and increasing bioinformatics accessibility will replace current methods in the near future.

Histopathological examination of aborted foetuses usually shows multifocal necrotising hepatitis, splenitis and placentitis; lesions typical for listeriosis. Therefore, histopathology should be combined with culture and/or molecular confirmation to identify the species of the disease causing agent (30, 45).

## 7 Antimicrobial susceptibility and treatment of *Listeria ivanovii* infection

Compared to the extensive literature available for *L. monocytogenes*, systematic data on the antimicrobial susceptibility of *L. ivanovii* remain limited. Most of the available evidence comes from sporadic human case reports, small collections of veterinary isolates, and limited comparative genomic analyses, rather than from structured surveillance programmes (26, 33, 48, 50). Published reports indicate that *L. ivanovii* isolates generally show susceptibility patterns similar to those of *L. monocytogenes*. Most investigated isolates are susceptible to *β*-lactams (e.g., penicillin G and ampicillin), aminoglycosides (when used in a synergistic combination), erythromycin, and trimethoprim-sulfamethoxazole (50). These findings are consistent with the conserved cell wall structure and target pathways across pathogenic *Listeria* spp. (26, 33, 50). However, intrinsic resistance to cephalosporins is a well-established characteristic of *Listeria* spp. including *L. ivanovii* (50).

Although *L. monocytogenes* is included in antimicrobial resistance (AMR) surveillance programmes in human and food safety contexts, *L. ivanovii* is rarely monitored separately. Available genomic analyses of *L. ivanovii* strains have not identified widespread acquired resistance determinants, although the number of sequenced isolates remains limited (33, 48). Comparative genomics suggests that the resistome of *L. ivanovii* is largely conserved and does not currently indicate the presence of major multidrug-resistant lineages.

A major limitation when interpreting the results of antimicrobial susceptibility testing (AST) for *L. ivanovii* is the absence of species-specific EUCAST or CLSI breakpoints. In routine laboratory practise, breakpoints defined for *L. monocytogenes* are often used by extrapolation. While this approach is pragmatic, differences between species cannot be entirely ruled out. Therefore, AST results for *L. ivanovii* should be interpreted with caution and ideally in the context of the clinical and epidemiological situation.

In cases of abortion in ruminants, antimicrobial therapy is often of limited success once placental colonisation has occurred. While treatment of affected dams can reduce systemic bacterial dissemination, it does not reliably prevent abortion once infection is established. In cases of septicaemia in lambs or other young animals, the recommended therapeutic approach based on classical listeriosis treatment principles is early administration of a high dose of penicillin or ampicillin often combined with an aminoglycoside (26, 50, 51).

Given the limited available data, a summary of reported antimicrobial susceptibility patterns of *L. ivanovii* is presented in Table 5.

**Table 5:** Reported antimicrobial susceptibility patterns of *L. ivanovii*^a^.

Antimicrobial class	Antibiotic tested	Reported susceptibility	AMR genes	Reference
Penicillins	Penicillin G, Ampicillin	Generally susceptible	No commonly reported acquired *β*-lactam resistance genes	(26, 33, 50)
Aminoglycosides	Gentamicin	Susceptible (in combination therapy)	No specific resistance determinants reported	(26, 50, 51)
Macrolides	Erythromycin	Most isolates susceptible	Rare resistance genes reported in *Listeria* spp.	(26, 48, 51)
Trimethoprim-SMX	TMP-SMX	Susceptible	No widespread resistance determinants reported	(33, 65)
Tetracyclines	Oxytetracycline	Variable susceptibility	Occasional *tet* genes in *Listeria* spp.	(48)
Cephalosporins	Cefotaxime, Ceftriaxone	Intrinsically resistant	Conserved intrinsic resistance	(50)
Fluoroquinolones	Ciprofloxacin, Enrofloxacin	Limited data; variable susceptibility	Rare mutations reported in *Listeria*	(48)
Glycopeptides	Vancomycin	Susceptible (human data)	No *van* genes reported	(33, 50)

a The data were derived from published clinical case reports, comparative genomic analyses and established antimicrobial susceptibility patterns of *Listeria* spp., particularly *L. monocytogenes*. Currently, no structured global surveillance programme specifically addresses *L. ivanovii*.

## 8 Prevention and control in animal husbandry

Given the predominantly animal-adapted ecology of *L. ivanovii*, prevention and control strategies should focus primarily on farm-level biosecurity, reproductive health management, and environmental hygiene. Unlike *L. monocytogenes*, large-scale foodborne outbreaks are not typical of *L. ivanovii*, so control measures shall focus on ruminant production systems (52). Although environmental contamination also plays an important role, the faecal-oral route is considered one of the main routes of infection transmission in ruminant herds. Furthermore, factors such as bedding, manure, water sources and feed can contribute to the persistence and circulation of bacteria within the herd (53, 54). Taxable data on *L. ivanovii* in silage are missing but the general ecology of *Listeria* spp. suggests that poor fermentation, elevated pH and aerobic spoilage increase the risk of contamination (53, 55). Thus, general preventive measures shall include proper silage fermentation (pH < 4.5), prevention of aerobic spoilage, regular cleaning and disinfection of lambing areas, manure management, rodent control, and separation of aborting animals. Environmental monitoring for *Listeria* spp. may support herd-level risk assessment, particularly on farms with recurrent abortion events (54). Abortion storms associated with *L. ivanovii* have primarily been documented in sheep flocks (5, 9, 39). Once abortion events are detected, the following control measures must be implemented rapidly: immediate isolation of affected ewes, removal and safe disposal of aborted foetuses and placental material, disinfection of contaminated areas, and use of personal protective equipment by farm workers. As antimicrobial therapy is not effective in preventing abortion management interventions are more important than pharmacological treatment.

Currently, there are no structured surveillance programmes that specifically target *L. ivanovii* in livestock populations. Most cases are identified incidentally during abortion diagnostics (26). Improved herd monitoring could entail the following: systematic laboratory examination of aborted foetuses for *L. ivanovii*, molecular confirmation of isolates and genomic analysis, and integration into regional or national veterinary surveillance frameworks. Given the probable underestimation of *L. ivanovii* prevalence, enhanced diagnostic resolution could clarify its true epidemiological burden in small ruminant production systems (48).

Appropriate sample collection is critical for reliable detection. Recommended specimens for outbreak investigations include placenta, foetal liver and spleen, abomasal contents, maternal vaginal swabs, and environmental samples, e.g., bedding, silage, water. Improper sampling and delayed submission may reduce culture sensitivity and contribute to diagnostic underrecognition (56).

Currently, there are no commercial vaccines specifically licenced against *L. ivanovii* in livestock. Control, therefore, relies entirely on management-based interventions. However, future research on host-adapted virulence factors, particularly those associated with LIPI-2, may provide insights for preventive strategies (48).

## 9 Knowledge gaps and future perspectives

Despite advances in molecular diagnostics and comparative genomics, significant knowledge gaps remain regarding *L. ivanovii*, particularly in veterinary contexts. Unlike *L. monocytogenes*, which is systematically monitored within food safety and public health surveillance systems across Europe and other regions (57, 58), *L. ivanovii* is not routinely reported in most monitoring frameworks (54). Most detections occur during investigations into abortions in small ruminants, and reporting often remains at the genus level. This hinders the accurate estimation of prevalence and geographic distribution (26, 54). Systematic identification of the species during abortion diagnostics would likely improve our understanding of epidemiology without implying an increased risk to public health.

Although recent whole-genome sequencing studies have increased the amount of genomic data available for *Listeria* spp., the number of publicly available *L. ivanovii* genomes remains limited, with most studies based on relatively small datasets, e.g., ten isolates especially compared to *L. monocytogenes,* for which several thousand genomes are available (48, 49). Although comparative genomic analyses confirm the presence of LIPI-2 and other virulence-associated determinants in *L. ivanovii*, strain diversity, population structure, and geographic clustering remain insufficiently characterised (33, 48). Increased genomic surveillance of veterinary isolates would clarify mechanisms of host adaptation and evolutionary relationships.

Although the ecology of *Listeria* spp. in farm environments has been extensively studied, species-specific ecological investigations focusing on *L. ivanovii* are missing. It is unclear whether *L. ivanovii* can persist long-term in silage, soil, or water independently of infected animals, or whether environmental contamination primarily reflects transient shedding (26). Longitudinal, farm-based studies incorporating molecular typing are required to address this knowledge gap.

Routine workflows often rely on genus-level detection or phenotypic identification. Without species-specific PCR or WGS confirmation, rare species such as *L. ivanovii* may be underreported (33, 35, 48). Improved molecular confirmation would likely increase detection accuracy, although current data do not suggest widespread circulation of this species.

Human infections with *L. ivanovii* are still uncommon (26, 52, 54). Currently, there is no evidence of sustained foodborne transmission comparable to that of *L. monocytogenes*. However, combining veterinary, environmental, and genomic data within a One Health framework could enhance our understanding of cross-species dynamics in the genus *Listeria*, particularly in agricultural ecosystems (48, 54).

## 10 Conclusion

*Listeria ivanovii* is a relatively uncommon but well-documented pathogen of veterinary importance primarily affecting ruminants. Available evidence consistently links it to reproductive disorders, particularly abortions in sheep and, less frequently, cattle. While large-scale epizootics or sustained interregional transmission have not been reported, sporadic outbreaks within flocks confirm that the bacterium can cause significant losses in farms.

Current knowledge suggests that the apparent rarity of *L. ivanovii* infections may be due to under-recognition in diagnosis rather than a true absence of the pathogen. Routine laboratory workflows often rely on genus-level identification, and species confirmation is not systematically performed in all abortion investigations. The increasing implementation of MALDI-TOF MS, species-specific PCR and whole-genome sequencing is likely to improve detection accuracy and provide a clearer epidemiological picture.

Genomic analyses support the concept of *L. ivanovii* as a host-adapted species with virulence determinants that differ from those of *L. monocytogenes*, particularly within the LIPI-2 pathogenicity island. However, current data do not suggest that it has broad public health relevance or circulates widely beyond livestock-associated environments.

In summary, *L. ivanovii* should be regarded as a specialised ruminant pathogen that requires accurate species-level identification in veterinary diagnostics. Improved surveillance, molecular confirmation and proportionate integration into existing *Listeria* monitoring frameworks will enhance our epidemiological understanding of the bacterium without overstating its impact.

## Footnotes

1
https://lpsn.dsmz.de/genus/listeria
